# MiR-218 Inhibits Invasion and Metastasis of Gastric Cancer by Targeting the Robo1 Receptor

**DOI:** 10.1371/journal.pgen.1000879

**Published:** 2010-03-12

**Authors:** Jun Tie, Yanglin Pan, Lina Zhao, Kaichun Wu, Jie Liu, Shiren Sun, Xuegang Guo, Biaoluo Wang, Yi Gang, Yongguo Zhang, Quanjiang Li, Taidong Qiao, Qingchuan Zhao, Yongzhan Nie, Daiming Fan

**Affiliations:** State Key Laboratory of Cancer Biology and Xijing Hospital of Digestive Diseases, Fourth Military Medical University, Xi'an, Shaanxi, China; Fred Hutchinson Cancer Research Center, United States of America

## Abstract

MicroRNAs play key roles in tumor metastasis. Here, we describe the regulation and function of miR-218 in gastric cancer (GC) metastasis. miR-218 expression is decreased along with the expression of one of its host genes, Slit3 in metastatic GC. However, Robo1, one of several Slit receptors, is negatively regulated by miR-218, thus establishing a negative feedback loop. Decreased miR-218 levels eliminate Robo1 repression, which activates the Slit-Robo1 pathway through the interaction between Robo1 and Slit2, thus triggering tumor metastasis. The restoration of miR-218 suppresses Robo1 expression and inhibits tumor cell invasion and metastasis *in vitro* and *in vivo*. Taken together, our results describe a Slit-miR-218-Robo1 regulatory circuit whose disruption may contribute to GC metastasis. Targeting miR-218 may provide a strategy for blocking tumor metastasis.

## Author Summary

MicroRNAs have been identified as playing important roles in tumor metastasis, but their impact on GC metastasis has been poorly explored. We have discovered miR-218, which functions as a suppressor of tumor metastasis and is correlated with clinical stage, lymph node metastasis, and prognosis in patients with GC. Our results show that miR-218 is part of a regulatory circuit involving the Slit-Robo1 pathway. In metastatic tumor cells, miR-218 was suppressed along with Slit3, one of its host genes. Meanwhile, Robo1, one of several Slit receptors, is upregulated in response to the decrease in miR-218, which in turn induced a reactive upregulation of the Slit-Robo1 pathway through an interaction with Slit2, thus facilitating tumor cell migration and invasion. Such findings not only provide new insights into the metastatic mechanisms in GC but also provide evidence for a novel miRNA–mediated regulatory mode of receptor signaling.

## Introduction

Advances in diagnostic and therapeutic approaches have led to excellent expectations for long-term survival for early gastric cancer (GC). However, the prognosis for advanced GC with extensive invasion and metastasis remains poor [1]. In order to metastasize, tumor cells must pass through a series of sequential and selective events, including detachment, migration, local invasion, angiogenesis, intravasation, survival in the circulatory system, extravasation, and regrowth in different organs. In the metastatic cascade, invasion of GC into the surrounding tissue is a crucial early step [2]–[4] **.** However, the mechanisms of invasion have not yet been fully elucidated.

A large number of microribonucleic acids (microRNAs or miRNAs) have been recently implicated in cancer metastasis [5], including miR-10b, miR-21, miR-126, miR-335, miR-373, miR-146, miR-520c, and miR-205 in breast cancer [6]–[11]; miR-224 and miR-21 in prostate cancer [12],[13]; miR-29c in nasopharyngeal carcinomas [14]; miR-10a, miR-222, miR-125b, miR-7, and miR-452 in urothelial carcinomas [15]; miR-182 in melanoma [16]; miR-92b and miR-9/9* in brain tumors [17]; and miR-21 in colorectal cancer [18]. However, very few miRNAs known to be involved in GC metastasis have been reported. miRNAs are naturally occurring, short, non-coding RNA molecules that negatively regulate gene expression [19]. In mammals, mature miRNAs are generated from pri-miRNAs and pre-miRNAs via sequential processing by Drosha and Dicer and are found in many organisms. They consist of 21–24 nucleotides, integrate into RNA-inducing silencing complexes, and pair with the 3′ untranslated regions (3′-UTR) of specific target messenger RNAs (mRNAs) to suppress translation or induce degradation of the target mRNAs [20]. Emerging evidence has revealed that miRNAs play key roles in various biological processes, including cell differentiation, proliferation, apoptosis, stress resistance, fat metabolism, tumorigenesis, and metastasis [21]–[23]. A better understanding of the changes in miRNA expression during GC invasion may lead to a better understanding of GC development, as well as possible improvements in the diagnosis and treatment of advanced GC.

In the present study, we established high (MKN28-M and SGC7901-M) and low invasive cell sublines (MKN28-NM and SGC7901-NM) using a repetitive transwell assay *in vitro*. We then examined the global miRNA expression profile in each cell subline using a miRNA microarray to identify differentially expressed miRNAs related to human GC invasion. In total, 45 miRNAs were shown to be differentially expressed in invasive vs. non-invasive GC cells. Among these, miR-218, a significantly downregulated miRNA in highly invasive cells, was shown to be closely correlated with GC tumorigenesis and metastasis in patients. More recently, a decrease in miR-218 has been reported in several kinds of solid tumors, including prostate cancer, GC, lung cancer, and cervical carcinoma [24]–[27], but this decrease in miRNA-218 was simply screened out as being one of the dozens of potential miRNAs of interest in the cancers described above. No further studies have been performed to assess the significance of miR-218 in tumor metastasis. Here, we have found that decreased miR-218 expression was correlated with advanced clinical stage, lymph node metastasis, and poor prognosis in patients, and re-expression of miR-218 in metastatic cells was able to inhibit migration, invasion, and metastasis formation both *in vitro* and *in vivo*. Using a bioinformatics search for miR-218 targets, we pinpointed the receptor Robo1 as miR-218's functional target, and we confirmed that the interaction between miR-218 and Robo1 was crucial to GC cell motility by demonstrating that there was an inverse correlation between miR-218 and Robo1 in GC cell lines as well as in GC patients. Furthermore, we discovered an intriguing negative feedback loop involving Slit, miR-218, and Robo1, in which miR-218 can be derived from either of two genes located in the introns of two distinct members of the Slit protein family. In addition, members of this family are ligands of the Robo1 receptor. We demonstrated that expression of the two miRNA precursor genes (miR-218-1 and miR-218-2) correlated with expression of the host genes (Slit2 and Slit3, respectively) and that the mature miR-218 was mainly derived from the miR-218-2 precursor, with a concomitant reduction of host Slit3 but not of Slit2 in metastatic GC cells. Thus, upregulation of Robo1 in response to the decrease in miR-218 induced a reactive upregulation of the Slit-Robo1 pathway through its interaction with Slit2, thus facilitating tumor cell invasion and metastasis. Our findings not only provide new insights into the metastatic mechanisms in GC, but they also revealed a novel regulatory mechanism of receptor signaling.

## Results

### Establishment and characterization of cell sublines with different invasive and metastatic potentials

**Figure 1:**
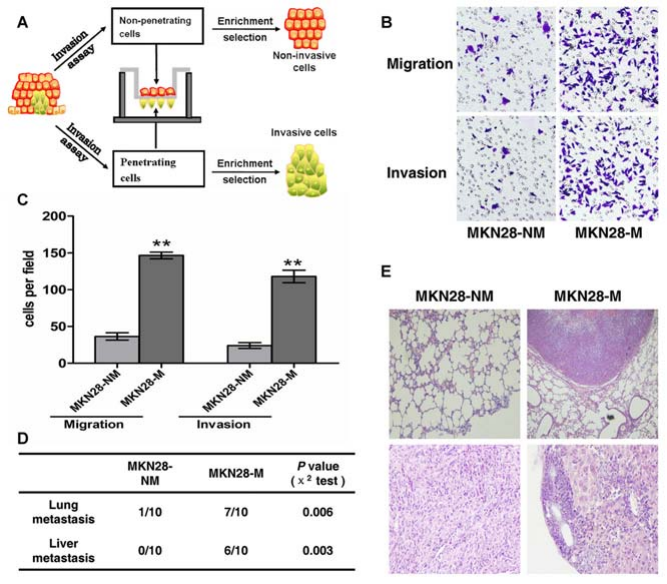
The metastatic characteristics of each cell subline. (A) General scheme of the establishment of invasive and non-invasive cell sublines derived from human GC cell lines. (B,C) *In vitro* migration and invasion activity of each cell subline**.** Migration and invasion activities were measured *in vitro* with transwell chambers, as described in Materials and Methods. Photos are representative fields of invasive cells on the membrane. Magnification, 100x. Bar graphs represent the average number of cells on the underside of the membrane ± SE. ** *P*<0.01 as compared with non-invasive cells, analyzed by *t*-test. (D and E) Metastasis potential of each cell subline *in vivo* (n = 10). (D) The incidence of metastasis in mice that received intravenous tail injections of each selected cell subline. (E) H&E staining of lungs and livers isolated from mice that received intravenous tail injections of MKN28-NM and MKN28-M cells, respectively. Magnification, 100x.

To establish the GC metastasis models, we created invasive and non-invasive cell sublines from the human GC cell lines SGC7901 and MKN28 using the repeated transwell approach (Figure 1A, see Materials and Methods). Briefly, a repetitive invasion assay was performed, and those cells that failed to invade the membranes and cells that had the ability to migrate through the collagen-coated membrane in all selection rounds were separated. After ten rounds of selection, we obtained invasive (MKN28-M and SGC7901-M) and non-invasive cell sublines (MKN28-NM and SGC7901-NM). The metastatic properties of each cell subline were then characterized *in vitro* and *in vivo*. As shown in Figure 1B and 1C, migration ability of MKN28-M cells was approximately 4-fold greater than that of MKN28-NM cells. Likewise, the invasive potential was about 5-fold greater for MKN28-M cells as compared to MKN28-NM cells. In the *in vivo* studies, tumor cell metastasis was observed in nude mice. As shown in Figure 1D and 1E, almost no metastatic GC cells were detected in the lungs or livers of nude mice at 10 weeks after injection of MKN28-NM cells, whereas most of the mice injected with MKN28-M cells displayed obvious lung or liver metastases. Similar results were observed for SGC7901-M and SGC7901-NM cells (data not shown). No significant differences in cell proliferation or cell-cycle distribution were observed among these cell sublines (Text S1, Figures S1 and S2).

### Identification of metastasis-related miRNAs by array-based hybridization

To identify miRNAs potentially involved in GC invasion, we examined global miRNA expression in each cell subline using the microRNA array (v.10.0, Exiqon, Vedbaek, Denmark), which consists of 847 capture probes for mature human miRNAs. The microarray results revealed that the expression of 124 miRNAs significantly differed between the highly invasive variant MKN28-M and the non-invasive cell subline MKN28-NM. Of these, 83 were upregulated and 41 were downregulated. Compared with SGC7901-NM, 62 miRNAs were differentially expressed in the SGC7901-M cell subline, including 47 downregulated and 15 upregulated miRNAs. In total, 11 miRNAs were found to be upregulated and 34 miRNAs were downregulated in both MKN28-M and SGC7901-M cells compared with those in the corresponding non-invasive sublines (Table S1).

**Figure 2:**
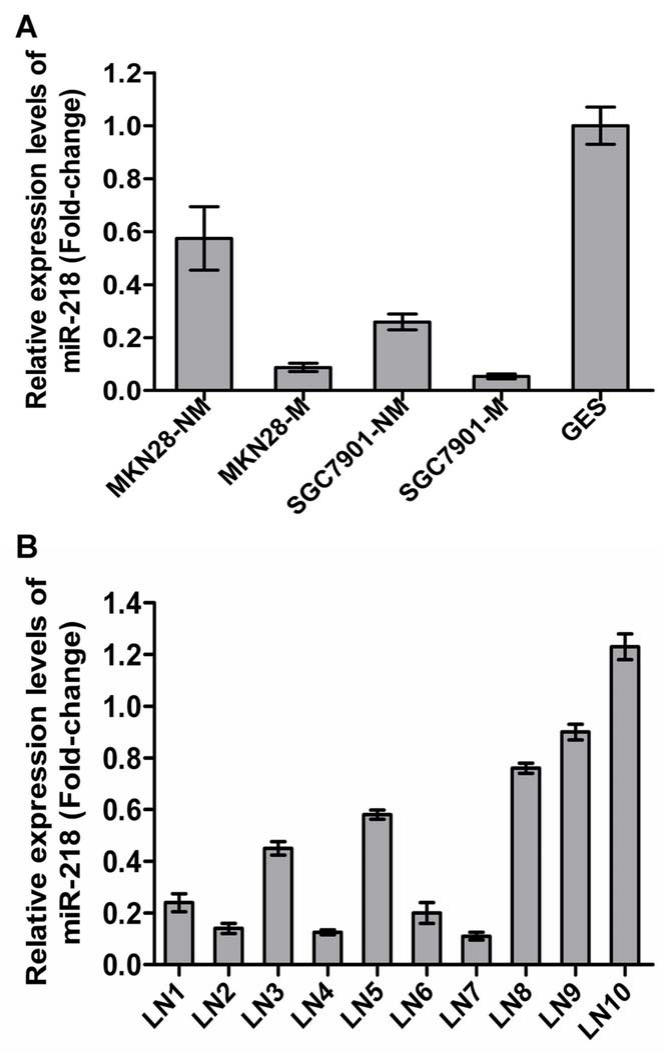
Validation of miR-218 expression in metastatic GC cells. The expression of miR-218 was investigated by qRT-PCR. (A) Each bar represents the relative fold change compared to GES cell lines. (B) Bars represent relative fold changes between primary GC and metastatic lymph nodes from the same patient. Each sample was analyzed in triplicate and was normalized to U6. Fold change was calculated by 2^−ΔΔCt^. The results were consistent with the microarray data.

Of the 45 differentially regulated miRNAs, miR-218 was one of those that displayed significantly differential expression. miR-218 has been reported to be downregulated in cervical cancer [25], GC [26], lung cancer [27] and prostate cancer [28], indicating possible involvement in both oncogenic transformation and tumor metastasis. However, miRNA-218 was only one of the many potential miRNAs of interest in cancers. In this work, miR-218 has been investigated in much greater detail. To validate the microarray results, we assessed miR-218 expression in the GC cell sublines previously mentioned and in the immortal gastric epithelial cell line GES using qRT–PCR [29]. miR-218 expression was significantly decreased in MKN28-M and SGC7901-M cells and was lower in all four GC cell sublines compared to immortalized human gastric epithelial GES cells (Figure 2A). Furthermore, we compared miR-218 expression in the primary GC tumor vs. the metastatic lymph nodes in 10 patients with stage III/IV GC using qRT–PCR. As shown in Figure 2B, mature miR-218 levels were significantly decreased in 7 out of 10 metastatic lymph nodes, indicating that miR-218 may play a causal role in GC metastasis.

### Decreased miR-218 expression in GC was associated with advanced clinical stage, lymph node metastasis, and poor patient prognosis

**Figure 3:**
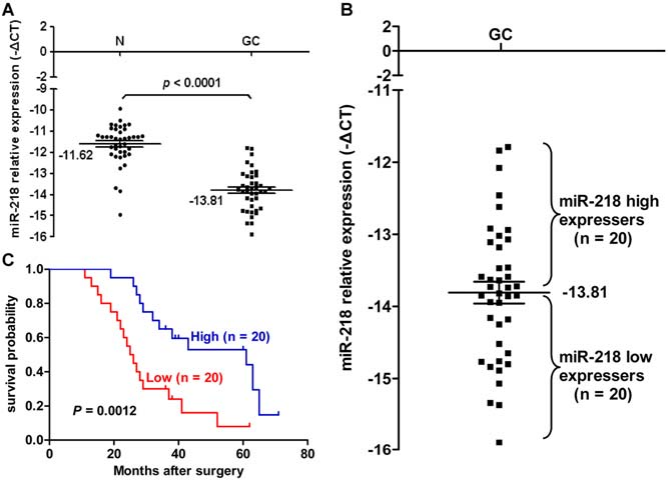
miR-218 expression in clinical GC specimens. (A) miR-218 was differentially expressed between GC and the corresponding non-neoplastic mucosa (N). The term -ΔCt was used to describe the expression level of miR-218 (−ΔCt = CtU6−CtmiR-218). A significant difference was detected in the mean value of miR-218 expression between these two groups (*P*<0.0001, t = 10.62, paired *t*-test). (B) The same GC samples as in (A) were divided into two groups according to the mean expression of miR-218 (mean, −13.81). Cases with levels of miR-218 below the mean were miR-218 low expressers (*n* = 20), and those with levels of miR-218 above the mean were miR-218 high expressers (*n* = 20). (C) Kaplan–Meier survival curve and log-rank test for GC patients between high and low miR-218 expressers. miR-218 expression demonstrated a significant relationship with patient survival (log-rank, *P* = 0.0012).

To determine the potential clinicopathological implications of altered miR-218 expression, we investigated the expression levels of miR-218 in 40 GC tissues (T) and non-tumor mucosa (N) b**y** qRT–PCR. The term -ΔCt was used to describe the expression level of miR-218. Consistent with the above data, the results verified that the miR-218 expression level in GC (-13.81±0.15, mean ± SE) tissues was significantly lower than that in non-neoplastic mucosa (-11.62±0.15, mean ± SE) (*P*<0.0001, t = 10.62, paired *t*-test) (Figure 3A). Correlations between the miR-218 expression level and clinicopathologic characteristics of GC are summarized in Table 1. Statistically significant associations between the miR-218 expression level and clinical stage and between the miR-218 expression level and GC metastasis were observed in this study. The median expression of miR-218 was −14.25±0.17 in the 22 cases with advanced stage (stage III and IV) disease, whereas the median expression was −13.27±0.20 (*P* = 0.0010, Mann-Whitney test) in the 18 cases with early-stage (stages I and II) disease. In the 29 cases of GC with lymph node metastasis, the median expression of miR-218 was −14.09±0.16, which was significantly lower than the median expression (−13.07±0.24) in the 11 non-metastatic GC cases (*P* = 0.0036). The expression of miR-218 in GC patients did not correlate with age, gender, tumor size, or cell differentiation. Moreover, we examined whether the level of miR-218 expression was associated with survival in patients with GC. Patients were subsequently divided into low expression (n = 20) and high expression groups (n = 20) based on miR-218 levels greater or less than the mean (−13.81) (Figure 3B). Kaplan–Meier survival analyses revealed that patients whose primary tumors displayed low expression of miR-218 had a shorter median survival time. The three-year survival rate of patients with low miR-218 expression was 30%, which was significantly lower than the survival rate in patients with high miR-218 expression (65%; *P* = 0.0012, log-rank test; Figure 3C).

**Table 1:** The relationship between clinicopathological parameters and miR-218 expression in primary gastric adenocarcinoma.

Variable	Number of cases	%	Median expression of miR-218(range)	*P*-value
Age (years)				
≥60	16	40%	−13.83±0.22	0.85
<60	24	60%	−13.79±0.21	
Gender				
Male	30	75%	−13.86±0.16	0.43
Female	10	25%	−13.66±0.38	
Tumor size				
≥5	26	65%	−13.91±0.20	0.36
<5	14	35%	−13.62±0.22	
Degree of differentiation				
well and moderately differentiated	18	45%	−13.77±0.23	0.7649
poorly differentiated	22	55%	−13.84±0.20	
TNM stage				
Stage I/II	18	45%	−13.27±0.20	0.0010^*^
Stage III/IV	22	55%	−14.25±0.17	
Lymph node status				
Metastasis	29	72.5%	−14.09±0.16	0.0036^*^
No metastasis	11	27.5%	−13.07±0.24	

### Ectopic expression of miR-218 inhibited tumor cell invasion and metastasis *in vitro* and *in vivo*

**Figure 4:**
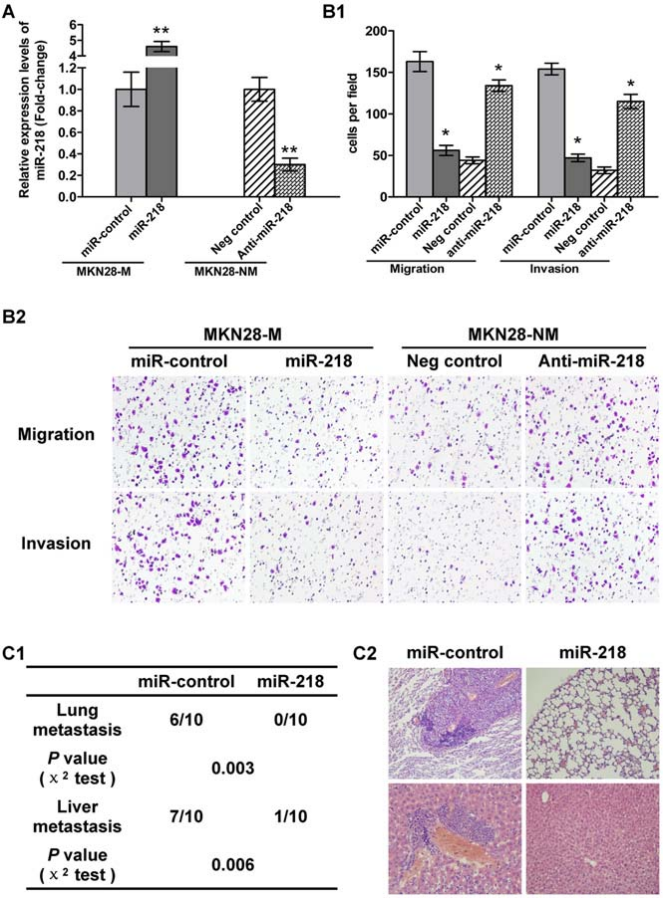
miR-218 suppressed tumor cell invasion and metastasis. (A) qRT–PCR analysis of miR-218 in MKN28-M cells transfected with the miR-218-expression vector or the miR-control vector and MKN28-NM cells transfected with anti-miR-218 or a negative control. (B) Cell invasion assay. (B1) Average number of invasive cells from three independent experiments ± SE. * *P*<0.05. (B2) Representative fields of invasive cells on the membrane. (C) *In vivo* metastasis assay. MKN28-M cells were transfected with the miR-218-expression vector or the miR-control vector and injected into nude mice via the tail vein, as described in Materials and Methods. Animals were killed 10 weeks after injection. (C1) Incidence of metastasis in mice. (C2) Representative H&E staining of lungs and livers isolated from mice that received injections of MKN28-M-miR-control or MKN28-M-miR-218 cells.

To study the role of miR-218 in GC metastasis, MKN28-M cells were transfected with pGenesil-1-miR-218 or a control vector expressing a nonspecific miRNA, cel-miR-67, using Lipofectamine 2000 (Invitrogen, Carlsbad, CA, USA). The cells were then selected with 400 mg/L G418 to generate MKN28-M-miR-218 and MKN28-M-miR-control stable cells. We found that ectopic expression of miR-218 resulted in an approximately three-fold reduction in migration and invasiveness. To determine whether the loss of miR-218 would promote the migration or invasion of cancer cells, we silenced miR-218 with an antisense oligonucleotide inhibitor in the MKN28-NM cell line, resulting in a three- to four-fold increase in cell migration and invasiveness (Figure 4A and 4B). To test if inhibition of tumor invasion by miR-218 is caused by impairing the invasive ability of tumor cells, we excluded the effect of miR-218 on the proliferation and cell cycle distribution of gastric cancer cells. Over-expression of miR-218 did not affect the proliferation and the cell cycle distribution of MKN28-M cells *in vitro* (Figure S5A and S5D). To further investigate the inhibition of *in vivo* tumor metastasis by miR-218, we implanted MKN28-M-miR-218 cells that were stably expressing miR-218 or control cells into nude mice through the lateral tail vein. Lung and liver metastasis of GC was apparent in mice injected with MKN28-M-miR-control cells. In contrast, few metastatic tumors were detected in mice injected with MKN28-M-miR-218 cells (Figure 4C). Furthermore, we simultaneously observed the growth of the primary tumors and the incidence of distant metastasis in the nude mice injected subcutaneously with MKN28-M-miR-218 cells or control cells. The results showed lung or liver metastasis was apparent in 3 out of 10 mice injected with MKN28-M-miR-control cells; in stark contrast, no metastasis were found in mice injected with MKN28-M-miR-218 cells (Figure S5E). These results are consistent with data obtained from tail vein assays that assess cancer metastasis and indicate that miR-218 has the ability to suppress metastasis without affecting cell proliferation.

### Robo1 was a direct functional target of miR-218 in GC metastasis

**Figure 5:**
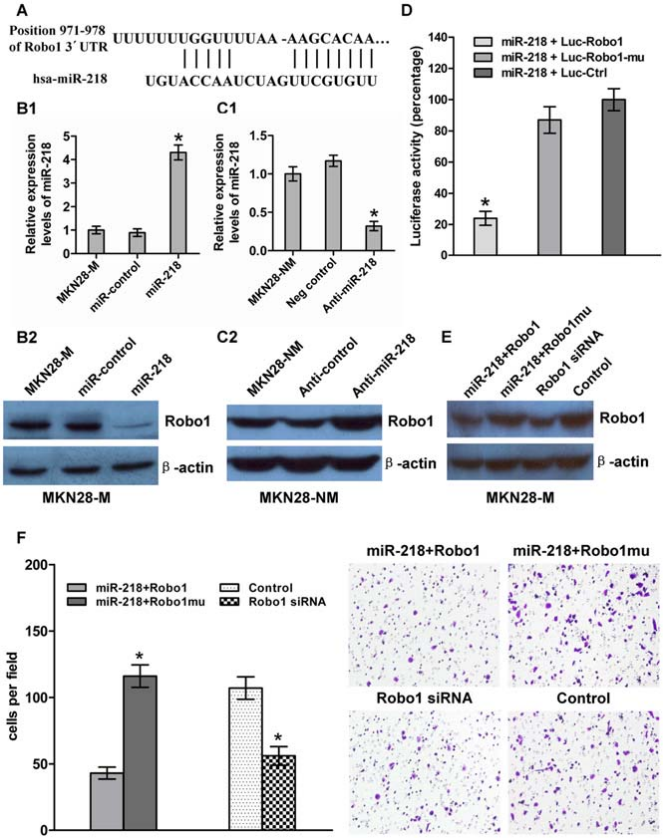
miR-218 targeted Robo1 by binding to its 3′-UTR. (A) The Robo1 3′-UTR was a potential target of miR-218. (B and C) miR-218 and Robo1 levels were analyzed by qRT–PCR and western blot, respectively. Robo1 levels decreased when miR-218 was upregulated in response to the miR-218-expression vector in MKN28-M cells, whereas the reverse was observed for Robo1 expression when miR-218 was knocked down in MKN28-NM cells. (D) MKN28-M cells were co-transfected with miR-218 and a luciferase reporter (Luc-Robo1) containing a fragment of the Robo1 3′-UTR harboring either the miR-218 binding site or a mutant (Luc-Robo1*-*mu) in which the first six nucleotides of the miR-218 binding site were deleted. A luciferase reporter construct engineered with a non-related fragment of cDNA was used as a negative control (Luc-control). The assays showed that luciferase activity in the Luc-Robo1 group was significantly decreased compared to the luciferase activity of the mutant and negative control groups. (E) MKN28-M-miR-218 cells, which stably over-expressed miR-218, were transiently transfected with a Robo1 expression construct or a Robo1 mutant construct lacking the miR-218 binding site. MKN28-M cells were transfected with Robo1 siRNA or a negative control siRNA. Western blot analysis for Robo1 showed that co-transfection of miR-218 and the Robo1 mutant construct produced higher levels of Robo1 protein than co-transfection of miR-218 and the Robo1 construct. Robo1 siRNA effectively reduced the amount Robo1 protein observed. (F) The cell invasion assay indicated that Robo1 mutant constructs could reverse the effect of miR-218-mediated suppression of cell invasion. Knockdown of Robo1 by siRNA in MKN28-M cells inhibited cell invasion. * *P*<0.05.

To assess how a low level of miR-218 expression contributes to the invasion and metastasis of GC, we searched for the potential regulatory targets of miR-218 using prediction tools, including miRanda, PicTar, and TargetScan. Although hundreds of different targets were predicted, those genes involved in migration or invasion may be the relevant targets with respect to the biological functions of miR-218. We then performed a functional classification of the predicted targets using the DAVID program (http://david.abcc.ncifcrf.gov/). Of these genes, Robo1 is regarded as a proto-oncogene and harbors migration-promoting activity [30]–[35]. Mertsch *et al.* demonstrated that Robo1 facilitates glioma cell migration mediated by Slit2 [36]. Schmid *et al.* found that breast tumor cell migration is induced by the Slit2-Robo1 interaction *in vitro* [37]. These findings suggest that Robo1 may be a target for miR-218. To further test our hypothesis, we analyzed the expression of miR-218 and Robo1 in GES and in non-invasive (MKN28-NM and SGC7901-NM) and invasive (MKN28-M and SGC7901-M) GC cells. The results showed a negative correlation between the levels of miR-218 and Robo1 mRNA in these cells (Figure S3A). Furthermore, we observed that Robo1 mRNA (Figure S3B) and protein (Figure 5B2) levels were decreased when miR-218 was expressed by pGenesil-1-miR-218 in MKN28-M cells (Figure 5B1). The reverse was observed for Robo1 expression when miR-218 was knocked down in MKN28-NM cells (Figure 5C1 and Figure 5C2). The inverse relationship between miR-218 and Robo1 expression was further confirmed by immunohistochemistry (Text S1) in 40 cases of gastric cancer, in matched adjacent normal tissues that were also used in clinicopathological studies, and in 29 matched metastases. The results show that Robo1 was upregulated in GC, especially in metastatic GC (Figure S4), in which miR-218 has a relatively low expression.

To obtain further direct evidence that Robo1 is a target of miR-218, we investigated the binding site of miR-218 in the 3′-UTR of Robo1 mRNA (Figure 5A). We constructed a luciferase reporter (Luc-Robo1) in which the nucleotides of the Robo1 3′-UTR complementary to miR-218 (nt 971–978) were inserted into the pMIR-REPORT miRNA expression reporter vector [38]. Correspondingly, we also generated both a mutant reporter (Luc-Robo1-mu), in which the first six nucleotides in the miR-218 seed-region complementary sites were deleted, and a control reporter, which contained a non-related fragment of cDNA (Luc-Ctrl). miR-218-expression plasmids were co-transfected with Luc-Robo1, Luc-Robo1-mu, or Luc-Ctrl into MKN28-M cells. The assays showed that the luciferase activity in the Luc-Robo1-transfected cells was significantly decreased compared to the luciferase activity in the mutant and negative control cells (*P*<0.05), suggesting that miR-218 reduced the luciferase activity of Luc-Robo1 but had no effect on Luc-Robo1-mu (Figure 5D). Therefore, we concluded that the inserted fragment of Robo1 (nt 971*–*978) was the target of miR-218.

Robo1 has been shown to be over-expressed in cancer cells and is known to promote tumor angiogenesis and metastasis via an interaction with Slit [39],[40]. To test whether Robo1 is functionally regulated by miR-218, we generated a Robo1 expression construct containing only a fragment of the predicted miR-218 binding site and Robo1 mutant expression vector entirely lacking the 3′-UTR. We also made the Robo1 siRNA. MKN28-M-miR-218 cells, which stably expressed miR-218 ectopically, were transiently transfected with the Robo1 construct or the mutant construct (with no miR-218 binding site), and MKN28-M cells were transfected with Robo1 siRNA or a negative control siRNA. MKN28-M-miR-218 cells transfected with the Robo1 mutant construct showed a 3.8-fold increase in invasion ability compared to cells transfected with the Robo1 construct. These results indicate that introduction of mutant Robo1 cDNA that lacked the miR-218 binding site into the miR-218-overexpressing cells reversed the effect of miR-218-mediated suppression of cell invasion. However, the effect of Robo1 was repressed by miR-218 in the presence of the Robo1 3′-UTR containing the miR-218 binding sites. Knockdown of Robo1 by siRNA in MKN28-M cells inhibited cell invasion, which fell to levels similar to those observed after transfection with the miR-218-expressing vector (Figure 5E and 5F). These observations suggest that miR-218 directly suppresses Robo1-mediated cell invasion.

### Slit2, but not Slit3, can interact with Robo1, enriched by the absence of miR-218, to promote GC invasion

**Figure 6:**
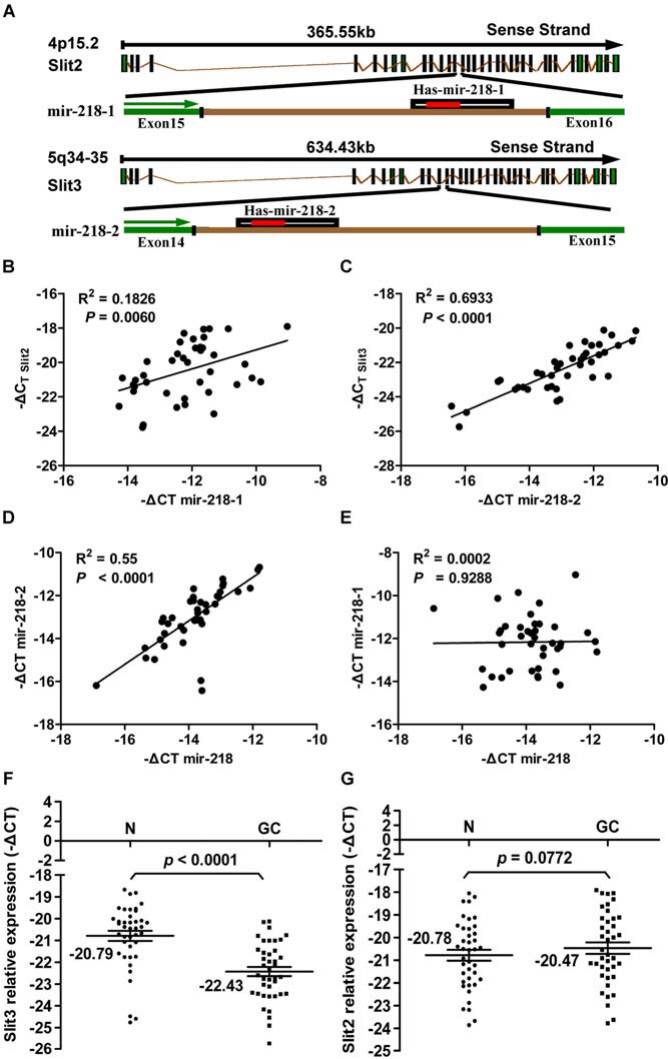
Results of the expression analysis of miR-218, miR-218-1, miR-218-2, Slit2, and Slit3 in 40 matched GC tumors and corresponding normal tissues via qRT–PCR. (A) Schematic representation of the miR-218 genomic locus hosted in the intron of Slit. Expression patterns of Slit2 with miR-218-1 (B) and Slit3 with miR-218-2 (C) exhibited a significant positive correlation, as did mature miR-218 with the miR-218-2 precursor (D), but not with miR-218-1 (E), in GC. A significant differential gene expression pattern was detected between normal and tumor samples with regard to Slit3 (*P*<0.0001, paired Student's *t*-test, Figure 6F), but not Slit2 (*P* = 0.0772, Figure 6G). Using relative quantification methods, the results were expressed as –ΔCt. The left and right lines of (F,G) represent the mean values for the normal and tumor groups, respectively.

Two types of miRNAs exist: intergenic and intronic. The former are located in non-coding regions between genes, and their corresponding pri-miRNAs are generally transcribed from their own promoters by RNA polymerase II. The latter are located within the introns of host genes, and their biogenesis is controlled by the host gene promoters [41],[42]. miR-218 is an intronic miRNA. Two genes code for mature miR-218, miR-218-1 and miR-218-2, which are located within intron 15 of Slit2 and intron 14 of Slit3, respectively (Figure 6A). The intronic location of the two miR-218 genes prompted us to ask whether miR-218-1 and miR-218-2 are transcribed together with their host gene mRNAs. To test this hypothesis, we used qRT-PCR to examine the expression of the miR-218-1 precursor, the miR-218-2 precursor, mature miR-218, Slit2 mRNA, and Slit3 mRNA in the GC tissues used in the survival analysis. Statistical analysis of the correlation coefficient of the qRT-PCR results revealed a significant positive correlation between the levels of Slit2 mRNA and miR-218-1 and between the levels of Slit3 mRNA and miR-218-2 (Figure 6B and 6C). These results indicate that the miR-218 coding genes, miR-218-1 and miR-218-2, are transcribed together with their host genes, Slit2 and Slit3, respectively. A significant positive correlation between the levels of miR-218 and miR-218-2 (Figure 6D) was seen in GC; however, no such correlation was seen between the levels of miR-218 and miR-218-1 (Figure 6E). These results indicate that downregulation of miR-218 in GC is promoted by a decrease in miR-218-2, but not in miR-218-1. Consistent with this conclusion, Slit3 expression was significantly reduced in GC (−22.43±0.21, mean ± SE) compared to normal gastric tissue (−20.79±0.23, mean ± SE), (*P*<0.0001, t = 7.67, paired *t*-test) (Figure 6F), whereas Slit2 expression was not significantly different (*P* = 0.0772, paired *t*-test) (Figure 6G). In summary, our experimental results suggest that significant upregulation of the Robo1 gene in response to removal of miR-218 may induce a subsequent upregulation of the Slit-Robo1 pathway through its interaction with Slit2, facilitating tumor cell migration and invasion.

## Discussion

To study a disease, it is vital to construct an ideal model. In the present study, we isolated invasive and non-invasive cell subpopulations from established human GC cell lines using the repeated transwell approach, which has been successfully applied in many studies investigating tumor metastasis [43]–[48]. The results of metastatic examination *in vitro* and *in vivo* showed that the established cell sublines had distinct invasive and metastatic capabilities. Here, we screened not only cell sublines derived from GC cell lines with high-invasive potential, but also those with low-invasive potential. With the exception of their metastatic abilities, the selected cell sublines were both quite similar, since they share the same genetic background. Since the major difference between the two types of sublines is metastatic capability, the genes that differ between them should correlate well with metastasis. Moreover, our method is able to distinguish invasion stages from metastasis and enables the study of specific steps in metastasis, which cannot be assessed in the live-animal model.

Recently, miRNAs have been reported to promote [49],[50] or suppress [51]–[54] tumor metastasis, providing a new perspective on the metastatic process. Nonetheless, the role of miRNAs in GC metastasis is lacking. In this report, we explored and obtained for the first time 45 metastasis-related miRNAs in GC based on a well-established metastasis cell model. The finding that miR-218 was downregulated in metastatic GC is intriguing, as decreased miR-218 levels have been reported in several types of solid tumors [24]–[27],[55], indicating that the loss of miR-218 may be a common event in tumorigenesis. In the present study, we focused on the effect of miR-218 on GC metastasis and demonstrated that miR-218 acts as a tumor suppressor in GC metastasis. Restoration of miR-218 reduced cell migration and invasion *in vitro* and tumor metastasis *in vivo*. To obtain stable cell lines that over-expressed miR-218, we transfected MKN28-M cells with miR-218 plasmids and screened by G418. We selected twelve cell colonies in the miR-218-transfected group and found 10 out of 12 colonies exhibited remarkably uniform, stable and high-level expression of miR-218. Furthermore, three randomly chosen monoclonal cell lines exhibited similar reduction in invasive ablility. However, plasmid transfection strategies often result in lower integration efficiency compared to viral expression leading to the possibility of stochastic selection of rare functionally heterogeneous variants from the initial bulk population. Therefore the future use of viral expression systems should create a more unbiased starting population to test our hypothesis.

As part of our research on how the loss of miR-218 affects GC metastasis, we demonstrated that Robo1 was a critical downstream target of miR-218. It is known that Robo is an axon guidance receptor for Slit and is conserved in animals ranging from fruit flies to mammals. In mammals, three Slit (Slit1–3) and four Robo (Robo1–4) genes have been described [56],[57]. The Slit–Robo interactions convey signals mediating repulsive cues on axons and growth cones during neural development and participate in T cell and monocyte chemotaxis [58]–[64]. As for other developmental pathways, aberrant expression of the Slit*-*Robo genes has been observed in a variety of tumor types [65]–[68]. For instance, in breast carcinoma tissue samples, Robo1 has been shown to be over-expressed, and it has been demonstrated to induce migration of breast cancer cell lines [37]. Slit2-Robo1 signaling facilitates glioma cell migration [36] and is involved in angiogenesis by increasing microvessel density and tumor mass in a tumor xenograft model [30]. Wang *et al.* demonstrated that the over-expression of Robo1 in new blood vessels in tumors induces cancer neovascularization and growth via an interaction between Robo1 and its ligand, Slit2. They also identified phosphoinositide-3-kinase (PI-3K) as a downstream effector of Slit2/Robo1 signaling. This suggests that there exists a Slit–Robo1–PI-3K cascade that could lead to the generation of phosphoinositol-3,4,5-triphosphate and the subsequent activation of small GTPases that mediate cell movement and remodeling of the actin cytoskeleton [30],[35]. In contrast, other studies have argued that the downregulation of Robo1 caused by deletions or epigenetic modifications may play a role in tumor progression [69]–[73]. We propose that a tissue-specific expression pattern exists for the Slit-Robo genes.

In the current study, we found that Robo1 was often expressed at high levels in invasive cells and at low levels in non-invasive cells, whereas miR-218 displayed the opposite expression pattern. When we transfected the miR-218-expression vector and inhibiting oligonucleotides into MKN28-M and MKN28-NM cells, respectively, an inverse expression pattern was observed between miR-218 and Robo1, that is, if miR-218 expression was high, Robo1 expression was low and vice versa. This result was further confirmed in clinical samples and in luciferase activity assays. We also noticed that induction of expression of Robo1 by the Robo1 mutant construct without the miR-218 binding site could reverse miR-218-mediated suppression of tumor cell invasion. In contrast, knockdown of Robo1 gene expression by RNAi had an effect on reducing tumor cell invasion similar to that of the restoration of miR-218, although Robo1 knockdown alone demonstrated a weak effect. This could be because Robo1 is not the only target of miR-218 that is relevant to tumor metastasis. These findings indicate that the invasion suppression effect of miR-218 is at least partly mediated through a decrease in Robo1 expression. This is also the first study to show that the tumor-associated gene Robo1 is negatively regulated by miR-218 via a specific target site (nt 971–978) within the 3′-UTR. The Robo1 receptor is crucial for the response to extracellular signals and cellular phenotypic changes; therefore, tight regulation of the Robo1 receptor by miR-218 may facilitate more robust signal transduction. Anti-Robo1 monoclonal antibody has been reported to be an effective treatment for Robo1-expressing cancers [30],[33]. In the present study, we found a new inhibitor of Robo1, miR-218, that may potentially be used to treat some types of cancer.

As mentioned above, Slit1, Slit2, and Slit3 comprise the Slit family of proteins. Although the genes overlap, their expression patterns and functions are distinct. The former two proteins are known to be involved in axon guidance and cell migration [37],[74], while Slit3 is involved in the development of organs and organ systems, including the diaphragm and the kidney [75]. In agreement with these data, we found that Slit2, but not Slit3, interacted with Robo1 to promote GC invasion.

In addition, we demonstrated that the miR-218 coding genes were located in and transcribed together with Slit genes, which were Robo1 ligands, thus creating a negative feedback loop that regulates Slit/Robo1 signaling. Sailen Barik demonstrated that an intronic miRNA, miR-338, silenced genes that are functionally antagonistic to its host gene product, thus creating a positive feedback loop that assists in the physiological role of the host gene [76]. However, a miRNA hosted in a ligand gene that simultaneously targets its corresponding receptor gene has never been reported. We have identified a negative regulatory loop involving the ligand (host gene), the intronic miRNA, and the receptor, in which the miRNA is co-transcribed with the ligand while receptor expression is repressed. The associations among ligand/receptor and intronic miRNA indicate that the early steps in the information flow may have built-in controls to limit excess signal propagation, which include a negative feedback loop to preserve homeostasis. This regulatory model, which is based on intronic miRNAs, is a novel mechanism of regulation in receptor signaling systems. Even though the full regulatory circuitry of miR-218 has yet to be completely elucidated, our study revealed a potential negative feedback pattern in which a miRNA was co-transcribed with the Slits ligand and repressed Robo1 receptor expression. This study not only provides new insights into the metastatic mechanism of GC but also generates a set of testable hypotheses that are helpful for understanding the miRNA-mediated regulation of cellular ligand/receptor interactions.

In conclusion, we have identified miRNAs that are aberrantly expressed in invasive GC cells compared with non-invasive GC cells. Here we have showed that when significantly downregulated, miR-218 promoted GC cell invasion and metastasis, at least in part via induction of Robo1. This result indicates that restoration of miR-218 may be a rational therapeutic strategy for the treatment of GC in the future. It remains to be investigated whether the other differentially expressed miRNAs found in this study also participate in GC metastasis. Importantly, our findings have implications for describing new mechanisms for miRNA-mediated regulation of receptor signaling.

## Materials and Methods

### Ethics statement

All experimental procedures were approved by the Institutional Review Board of the Fourth Military Medical University. Written informed consent was obtained for all patient samples. Animal experiments were performed with the approval of the Institutional Committee for Animal Research and in conformity with national guidelines for the care and use of laboratory animals.

### Cell culture

The human GC cell lines MKN28 and SGC7901 were routinely maintained in RPMI-1640 medium (GIBCO, Carlsbad, CA, USA) supplemented with 10% fetal bovine serum, 100 U/ml of penicillin sodium, and 100 µg/ml of streptomycin sulfate at 37°C in a humidified air atmosphere containing 5% carbon dioxide. Throughout the experiment, cells were used in the logarithmic phase of growth.

### Isolation of invasive and non-invasive cell sublines using transwell chambers

Six-well polycarbonate transwell membrane inserts with 8-µm pores (Corning, USA) were used to isolate cell sublines with different levels of invasiveness from the cultured MKN28 cell line. First, cells that were serum-starved for 24 h were suspended in serum-free RPMI-1640 to a final cell density of 5×10^5^ cells/mL. A 1 mL cell suspension was seeded into the top chamber, which was coated with 200 mg/mL of Matrigel (BD Biosciences, San Jose, CA, USA), and the lower well beneath the polycarbonate membranes was filled with 2.5 mL RPMI-1640 medium supplemented with 20% bovine serum to create a chemotactic gradient to stimulate penetration of the cells. Following incubation for 24 h at 37°C, the invasive cells on the underside of the membrane and the non-invasive cells on the top of the membrane were harvested aseptically and were expanded for selection. Via ten-round selection, the cell subline that failed to invade through the membranes in all selection rounds was designated as MKN28-NM, and the subline that was able to migrate through the membranes was designated as MKN28-M. We also obtained two cell sublines, SGC7901-M and SGC7901-NM, derived from the SGC7901 GC cell line, using the same method (Figure 1).

### *In vitro* migration and invasion assays

A 24-well transwell plate (8-µm pore size, Corning, USA) was used to measure each cell line's migratory and invasive ability. For transwell migration assays, 2.5×10^4^ cells were plated in the top chamber lined with a non-coated membrane. For invasion assays, chamber inserts were coated with 200 mg/mL of Matrigel and dried overnight under sterile conditions. Then, 5×10^4^ cells were plated in the top chamber. In both assays, cells were suspended in medium without serum or growth factors, and medium supplemented with serum was used as a chemoattractant in the lower chamber. After incubation at 37°C for 24 h, the top chambers were wiped with cotton wool to remove the non-migratory or non-invasive cells. The invading cells on the underside of the membrane were fixed in 100% methanol for 10 min, air-dried, stained in 0.1% crystal violet, and counted under a microscope. The mean of triplicate assays for each experimental condition was used.

### Experimental metastasis

To produce experimental metastasis, cells were washed and resuspended in PBS. Five-week-old BALB/C-nu/nu nude mice obtained from the Shanghai Laboratory Animal Center of China were injected into the lateral tail vein, and the animals were maintained in a sterile animal facility. Each tumor cell subline was injected into ten mice. After ten weeks, the mice were killed, and the lungs and liver were examined for metastases. Tumor tissues derived from various organs were dissected and examined histologically. The experiments were repeated two to three times.

### miRNA microarrays

Total RNA was extracted from each cell subline using the miRVana miRNA Isolation Kit (Ambion Inc., Austin, TX, USA) according to the manufacturer's instructions. The purity and quantity of the isolated RNAs were assessed using 1% formaldehyde-agarose gel electrophoresis and spectrophotometry (Bio-Rad, Hercules, CA, USA). We then submitted the samples to KangChen-Biotech (Shanghai, China) for array hybridization on a miRCURY LNA microRNA array (v.10.0, Exiqon, Vedbaek, Denmark). Each microarray chip was hybridized with a single sample labeled with either Cy3 or Cy5. Background subtraction and normalization were performed. We selected miRNAs whose expression levels between invasive cell sublines and non-invasive cell sublines differed by at least 1.5-fold.

### Clinical samples

Forty patients (30 males and 10 females) who had undergone gastrectomy with lymph node dissection for gastric carcinoma at Xijing Hospital between March and September of 2003 were included in the study (Table S2). The patients ranged in age from 26 to 77 years (median 54.13 years). None of the patients received preoperative chemotherapy. The resected specimens were histologically examined by H&E staining. The primary tumor tissues and corresponding non-tumor mucosa and lymph nodes were collected from each patient immediately after surgical removal and snap-frozen in liquid nitrogen until further use. Total RNA from the frozen tissues was isolated with Trizol (Invitrogen, Carlsbad, CA, USA) according to the manufacturer's instructions.

### Quantitative real-time RT–PCR (qRT–PCR)

Total RNA was extracted with suitable reagents. The TaqMan stem-loop RT-PCR method was used to assess the expression of miRNAs with kits from Applied Biosystems (Foster City, CA, USA). SYBR green real-time RT-PCR was performed to detect Slit2, Slit3, and Robo1. All RT-PCR experiments were performed on a Chromo4 Real-Time PCR Detection System (Bio-Rad, Hercules, CA, USA). The primers for miR-218 and its precursors were obtained from Applied Biosystems(Foster City, California, USA)and Eurogentec North America, Inc (Flintkote Avenue, San Diego, California, USA), respectively. The primers for Slit2, Slit3, and Robo1 were designed to produce amplicons that were 76–150 bp in length and with an annealing temperature of approximately 60°C using Primer Premier v5.0 Software. Data are presented as fold differences relative to either 18S for Slit2, Slit3, and Robo1 or U6 for miRNA based on calculations of 2^−ΔΔCt^. All primer sequences in this study are listed in Table S3.

### Construct design and cell transfections

#### miR-218-expressing vector

The precursor sequence of miR-218 (110 bp, MI0000295) generated by annealing and primer extension with miR-218-precursor-F and miR-218-precursor-R (Table S3) was digested with BamHI and HindIII and cloned into the BamHI-HindIII fragment of the pGenesil-1 vector. A construct including the nonspecific miRNA cel-miR-67 (99 bp, MI0000038) was used as a negative control.

#### Luc-Robo1 vector

The Robo1 3′-UTR containing the predicted miR-218 binding site was amplified by RT-PCR from the total RNA of cultured MKN28 cells and was cloned into the pCR2.1-TOPO vector (Invitrogen, Carlsbad, CA, USA). The pCR2.1-TOPO-Robo1 3′-UTR construct was digested with SpeI and HindIII. The resulting fragment was subcloned into the SpeI and HindIII sites of the pMIR-REPORT miRNA expression reporter vector (Applied Biosystems). The first six nucleotides complementary to the miR-218 seed-region were deleted from the mutant constructs using the QuikChange Site-Directed Mutagenesis Kit (Stratagene) according to the manufacturer's protocol.

#### Robo1-expressing vector with or without miR-218 binding sites

Full-length Robo1 cDNA that entirely lacks the 3′-UTR (Clone ID: 9057080) was purchased from Open Biosystems (USA) and was subcloned into the eukaryotic expression vector pcDNA3.1(+) to generate the Robo1 mutant expression vector. A Robo1-expressing vector was constructed by inserting the fragment of the predicted miR-218 binding site into the Robo1 mutant expression vector. Robo1 siRNA and negative control oligonucleotides were purchased from GenePharma (Shanghai, China).

#### Cell transfections

MKN28-M cells were transfected with the miR-218-expressing vector or the control vector expressing a nonspecific miRNA, cel-miR-67, using Lipofectamine 2000 (Invitrogen), and were selected with 400 mg/L G418 to generate two stable monoclonal cell lines (a miR-218 stable cell line, MKN28-M-miR-218, and a control stable cell line, MKN28-M-miR-control). The oligonucleotides comprising the miR-218 inhibitor and the mismatched sequence negative control were purchased from Ambion Inc. and were transfected into MKN28-NM cells using Oligofectamine (Invitrogen). The Robo1-expressing vector with or without the miR-218 binding sites was transfected into MKN28-M-miR-218 cells, and Robo1 siRNA was transfected into MKN28-M cells**.**

#### Luciferase assay

The pMIR-REPORT β-galactosidase control vector and Luc-Robo1, Luc-Robo1-mu, or Luc-control were co-transfected into MKN28-M-miR-218 cells. Lysates were prepared at 48 h post-transfection. Luciferase activity was measured using the Dual-Light luminescent reporter gene assay (Applied Biosystems). All measurements were normalized to β-galactosidase activities to correct for variations in transfection efficiencies and for non-miR-218-specific effects of miRNA transfection on enzymatic activity.

#### Western blot

Cellular proteins were extracted and separated in SDS-PAGE gels, and western blot analyses were performed according to standard procedures. Western blotting of β-actin on the same membrane was used as a loading control. The antibodies used were anti-Robo1 (SC-25672) and anti-β-actin (sc-47778), both from Santa Cruz Biotechnology (CA, USA).

#### Statistical analyses

All data are presented as means ± SE and were analyzed using Prism 5.0 software (GraphPad). The significance of the observed differences was determined with the Student's *t*-test or the χ2 test. The relationships among the miR-218-1 precursor, the miR-218-2 precursor, mature miR-218, Slit2 mRNA, and Slit3 mRNA were analyzed by correlation coefficients and linear regression analysis. *P*<0.05 was considered statistically significant. * *P*<0.05; ** *P*<0.01.

## Supporting Information

Figure S1 No significant difference in the proliferation rate was observed in the three cell sublines. (A) Proliferation rates of the cell sublines were detected by the MTT assay. (B) Tumor volume growth curves for each cell subline are shown. Tumor sizes were measured using calipers. Tumor volume was calculated using the formula (length × width^2^)/2. (*n* = 5, paired Wilcoxon test, *P*>0.05). (C) On day 37, all tumors were collected to measure tumor weights (*P*>0.05; *n* = 5). (D) Photos of tumors 37 days after injection with MKN28, MKN28-NM, or MKN28-M cells.
(6.14 MB TIF)

Figure S2 Cell-cycle analysis of three established cell sublines. (A) Representative flow cytometry results for each cell subline. (B) Cell-cycle distribution (*P*>0.05; *n* = 3).
(2.82 MB TIF)

Figure S3 qRT-PCR analysis of the relative expression of miR-218 and Robo1. (A) Expression of miR-218 and Robo1 were reversed in invasive (MKN28-M and SGC7901-M) and non-invasive GC cells (MKN28-NM and SGC7901-NM) compared with GES cells. (B) Robo1 mRNA levels decreased when miR-218 was upregulated in response to transfection of MKN28-M cells with miR-218-expressing vector.
(2.56 MB TIF)

Figure S4 Immunohistochemical analysis of Robo1. (A-F) represent normal and tumor tissues taken from the same patient and processed in the same way using paraffin sectioning. (A,C,E) H&E staining of normal gastric mucosa, primary gastric cancer, and the ovarian metastasis from gastric cancer. Magnification, 100×. (B,D,F) Robo1 in normal gastric mucosa, primary gastric cancer, and ovarian metastasis from gastric cancer (serial section adjacent to the H&E-stained specimen). Magnification, 100×. Robo1 was expressed at low levels in the gastric epithelial cells of normal tissues and was expressed at increased levels in gastric cancer tissues, especially in metastatic tumor tissues.
(9.91 MB TIF)

Figure S5 miR-218 has the ability to specifically suppress metastasis without affecting cell proliferation. (A) MTT assay of the effects of miR-218 on proliferation of MKN28-M cells. No significant difference in the proliferation rate was found between MKN28-M-miR-218 cells stably over-expressing miR-218 and control cells. (B) MKN28-M-miR-218 and control cells were subcutaneously injected into nude mice. Growth curves of primary gastric cancers formed by MKN28-M-miR-218 cells or control cells are shown. Tumor sizes were measured using calipers. Tumor volume was calculated using the formula (length × width^2^)/2. Each data point represents the mean ± standard error (*n* = 10; *P*>0.05). (C) Median tumor weight at day 72. Data are presented as mean ± standard error (*n* = 10; *P*>0.05). (D) Cell cycle distribution (*P*>0.05; *n* = 3). (E) Representative H&E staining of lungs and livers isolated from mice that received injections of MKN28-M-miR-control or MKN28-M-miR-218 cells. Magnification, 200×.
(3.55 MB TIF)

Table S1 Differentially expressed miRNAs in highly invasive GC cells versus non-invasive GC cells.
(0.10 MB DOC)

Table S2 Clinicopathologic features in 40 tumor samples.
(0.25 MB DOC)

Table S3 Primer sequences used in the study.
(0.04 MB DOC)

Text S1 Supplementary methods.
(0.05 MB DOC)
