# Supplementary figures and images for: MiR-218 Inhibits Invasion and Metastasis of Gastric Cancer by Targeting the Robo1 Receptor
Source: PLoS Genet. 2010 Mar 12;6(3):e1000879. doi: 10.1371/journal.pgen.1000879 (PMC2837402; doi:10.1371/journal.pgen.1000879)

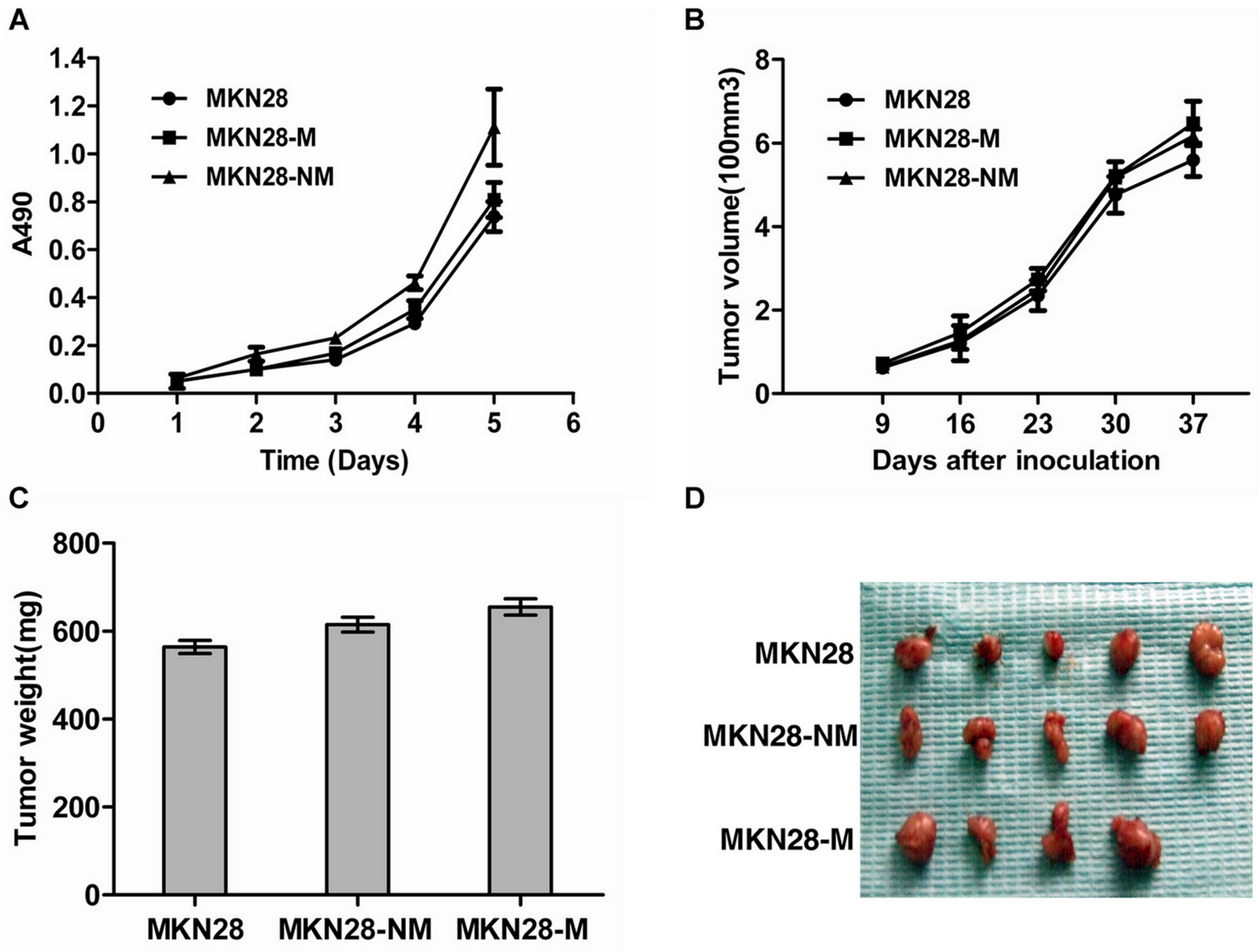

Supplement: Figure S1 — No significant difference in the proliferation rate was observed in the three cell sublines. (A) Proliferation rates of the cell sublines were detected by the MTT assay. (B) Tumor volume growth curves for each cell subline are shown. Tumor sizes were measured using calipers. Tumor volume was calculated using the formula (length × width2)/2. (n = 5, paired Wilcoxon test, P>0.05). (C) On day 37, all tumors were collected to measure tumor weights (P>0.05; n = 5). (D) Photos of tumors 37 days after injection with MKN28, MKN28-NM, or MKN28-M cells. (6.14 MB TIF) [file pgen.1000879.s001.tif]

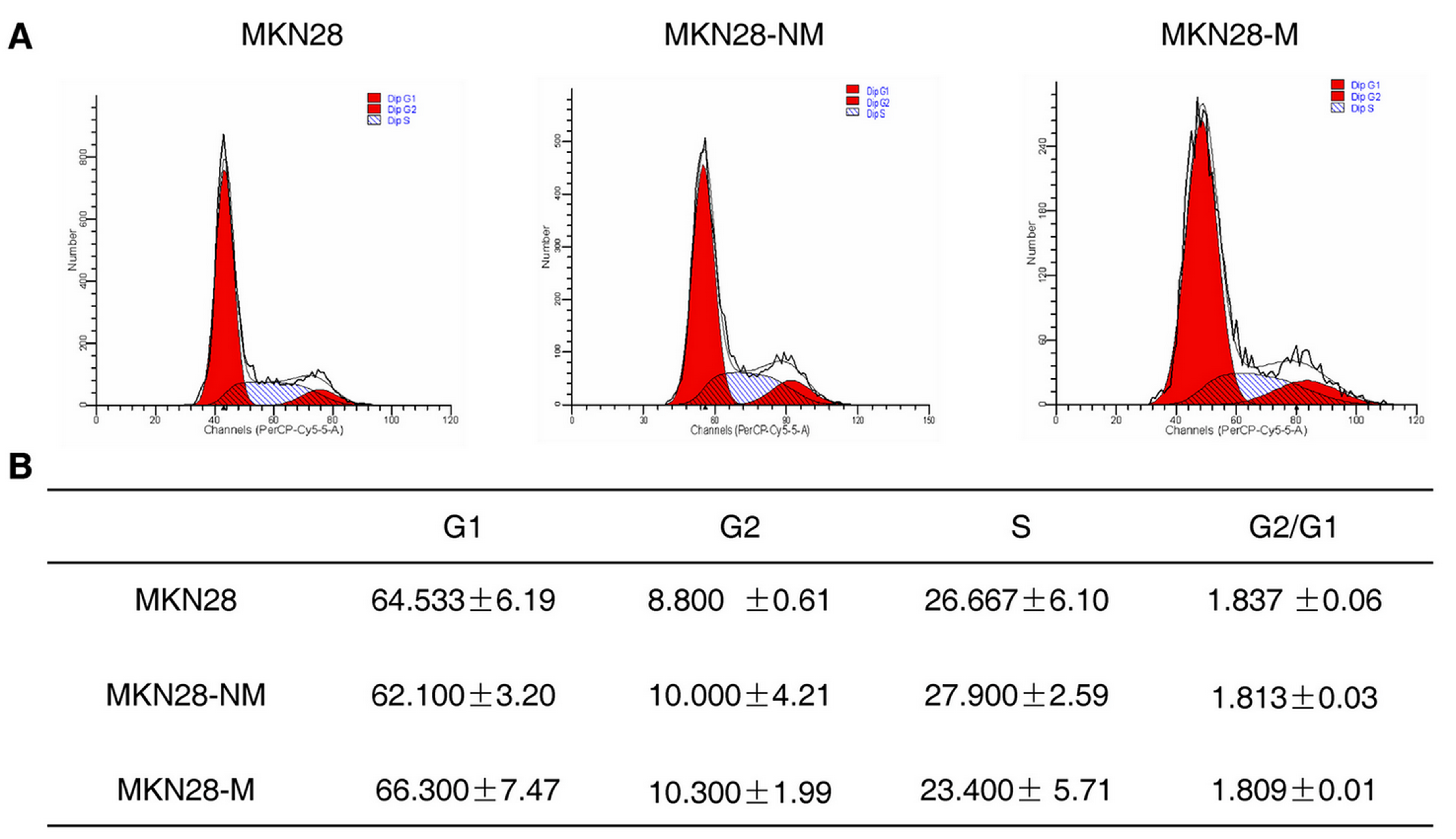

Supplement: Figure S2 — Cell-cycle analysis of three established cell sublines. (A) Representative flow cytometry results for each cell subline. (B) Cell-cycle distribution (P>0.05; n = 3). (2.82 MB TIF) [file pgen.1000879.s002.tif]

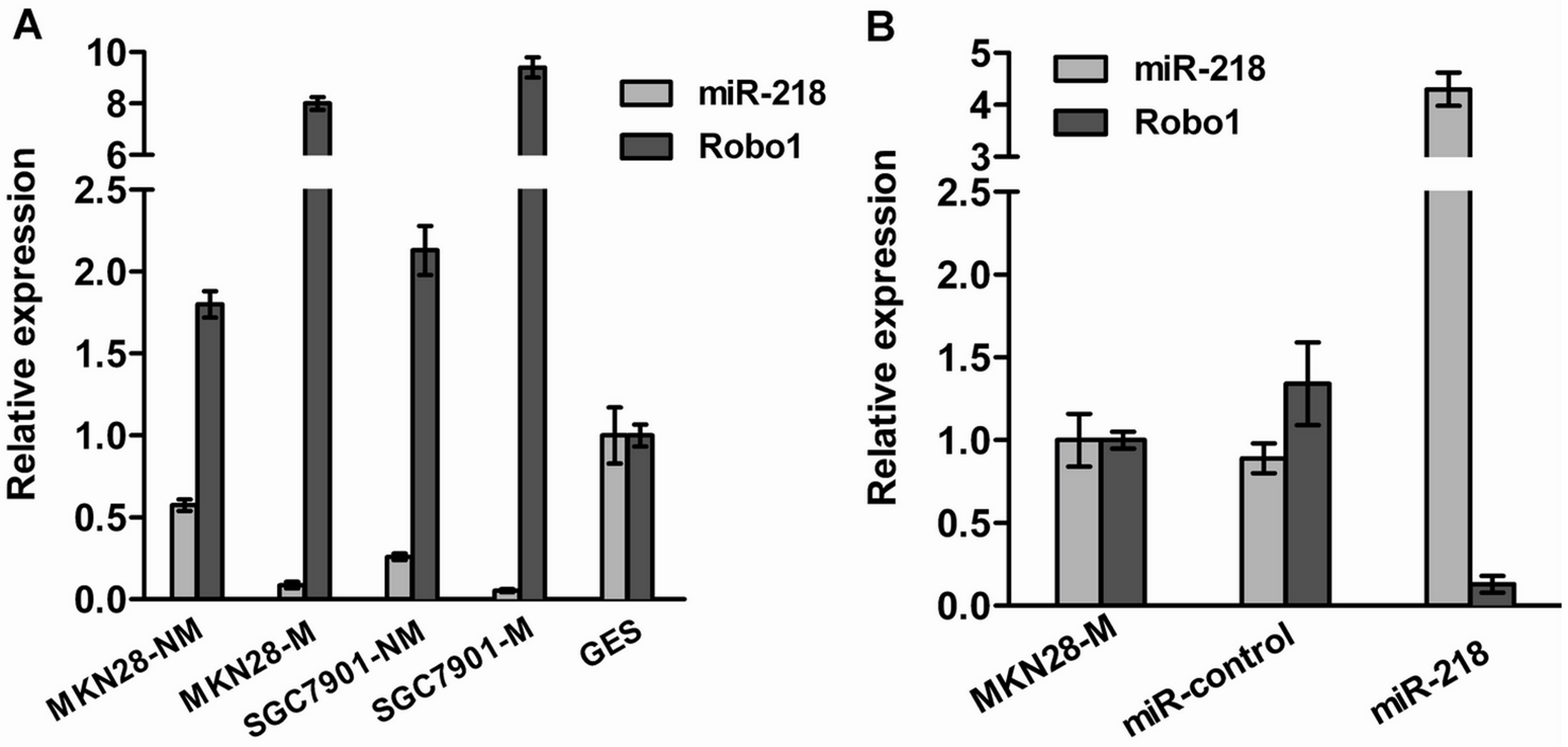

Supplement: Figure S3 — qRT-PCR analysis of the relative expression of miR-218 and Robo1. (A) Expression of miR-218 and Robo1 were reversed in invasive (MKN28-M and SGC7901-M) and non-invasive GC cells (MKN28-NM and SGC7901-NM) compared with GES cells. (B) Robo1 mRNA levels decreased when miR-218 was upregulated in response to transfection of MKN28-M cells with miR-218-expressing vector. (2.56 MB TIF) [file pgen.1000879.s003.tif]

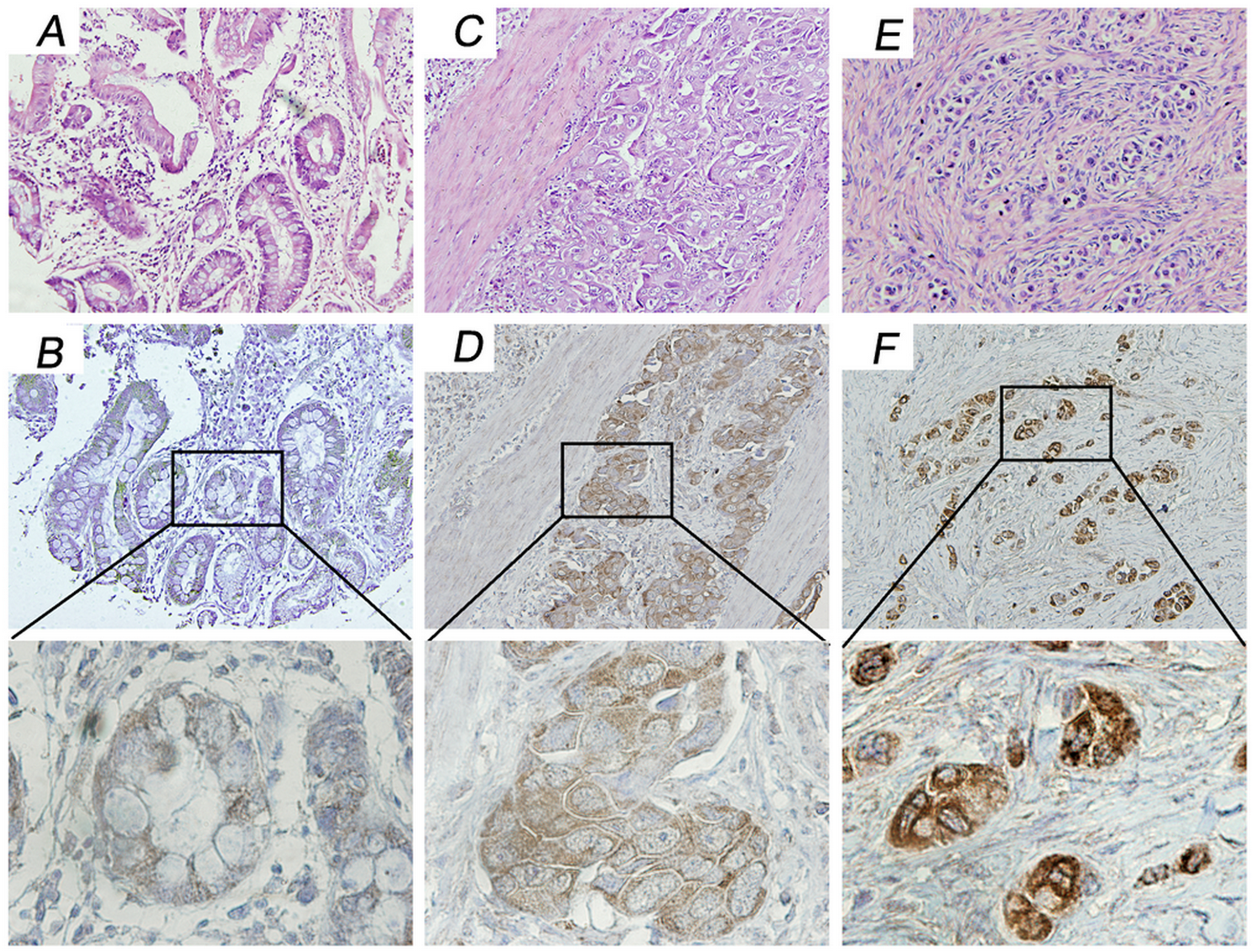

Supplement: Figure S4 — Immunohistochemical analysis of Robo1. (A-F) represent normal and tumor tissues taken from the same patient and processed in the same way using paraffin sectioning. (A,C,E) H&E staining of normal gastric mucosa, primary gastric cancer, and the ovarian metastasis from gastric cancer. Magnification, 100×. (B,D,F) Robo1 in normal gastric mucosa, primary gastric cancer, and ovarian metastasis from gastric cancer (serial section adjacent to the H&E-stained specimen). Magnification, 100×. Robo1 was expressed at low levels in the gastric epithelial cells of normal tissues and was expressed at increased levels in gastric cancer tissues, especially in metastatic tumor tissues. (9.91 MB TIF) [file pgen.1000879.s004.tif]

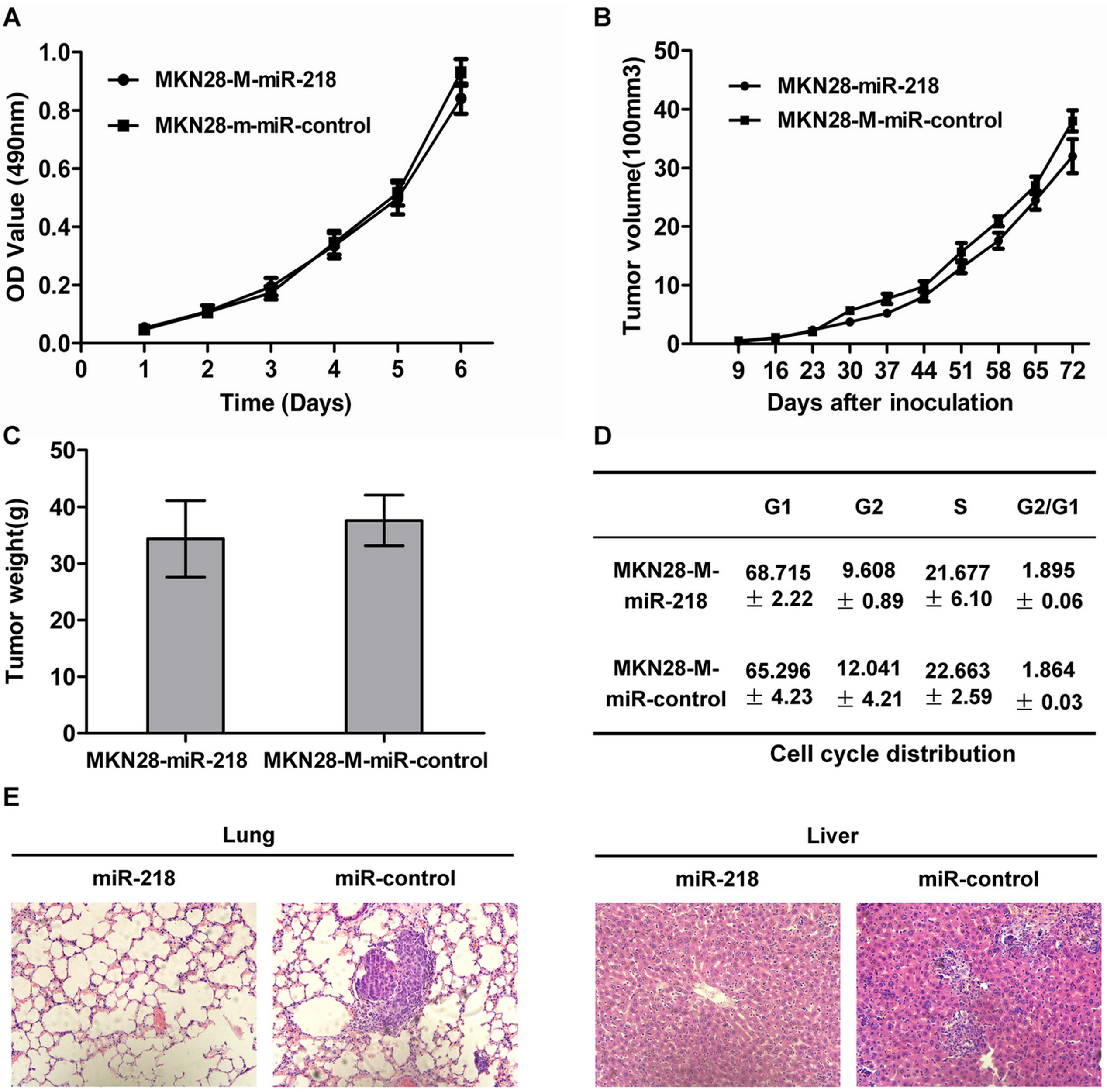

Supplement: Figure S5 — miR-218 has the ability to specifically suppress metastasis without affecting cell proliferation. (A) MTT assay of the effects of miR-218 on proliferation of MKN28-M cells. No significant difference in the proliferation rate was found between MKN28-M-miR-218 cells stably over-expressing miR-218 and control cells. (B) MKN28-M-miR-218 and control cells were subcutaneously injected into nude mice. Growth curves of primary gastric cancers formed by MKN28-M-miR-218 cells or control cells are shown. Tumor sizes were measured using calipers. Tumor volume was calculated using the formula (length × width2)/2. Each data point represents the mean ± standard error (n = 10; P>0.05). (C) Median tumor weight at day 72. Data are presented as mean ± standard error (n = 10; P>0.05). (D) Cell cycle distribution (P>0.05; n = 3). (E) Representative H&E staining of lungs and livers isolated from mice that received injections of MKN28-M-miR-control or MKN28-M-miR-218 cells. Magnification, 200×. (3.55 MB TIF) [file pgen.1000879.s005.tif]
