# Supplementary material for: MiR-218 Inhibits Invasion and Metastasis of Gastric Cancer by Targeting the Robo1 Receptor
Source: PLoS Genet. 2010 Mar 12;6(3):e1000879. doi: 10.1371/journal.pgen.1000879 (PMC2837402; doi:10.1371/journal.pgen.1000879)
Supplement: Table S1 — Differentially expressed miRNAs in highly invasive GC cells versus non-invasive GC cells. (0.10 MB DOC) [file pgen.1000879.s006.doc]

**Table S1.** Differentially expressed miRNAs in highly invasive GC cells versus non-invasive GC cells.

| No | Probe ID | Metastasis-miRNA | Expression in metastatic GC | Fold change (MKN28-M /MKN28-NM) | Fold change (SGC7901-M /SGC7901-NM) |
| --- | --- | --- | --- | --- | --- |
| 1 | 32884 | hsa-miR-342-3p | Up | 4.2294416 | 2.6392544 |
| 2 | 11061 | hsa-miR-329 | Up | 6.4149669 | 2.408681 |
| 3 | 17393 | hsa-miR-603 | Up | 4.2812843 | 2.1775587 |
| 4 | 17474 | hsa-miR-421 | Up | 3.4436061 | 1.7375701 |
| 5 | 42794 | hsa-miR-489 | Up | 3.2714303 | 1.7325632 |
| 6 | 17573 | hsa-miR-625 | Up | 1.9181312 | 1.7113464 |
| 7 | 11138 | hsa-miR-506 | Up | 2.3739594 | 1.6965787 |
| 8 | 11104 | hsa-miR-422a | Up | 2.0420028 | 1.6557034 |
| 9 | 42746 | hsa-miR-647 | Up | 2.5906276 | 1.5688161 |
| 10 | 42815 | hsa-miR-218-2* | Up | 2.2048761 | 1.5464648 |
| 11 | 42446 | hsa-miR-576-5p | Up | 2.0246328 | 1.5138727 |
| 12 | 42898 | hsa-miR-124* | Down | 0.1330953 | 0.0024477 |
| 13 | 4610 | hsa-miR-126 | Down | 0.3449926 | 0.0982179 |
| 14 | 11018 | hsa-miR-218 | Down | 0.1532823 | 0.1283594 |
| 15 | 42524 | hsa-miR-21* | Down | 0.0620057 | 0.1733466 |
| 16 | 42739 | hsa-miR-339-5p | Down | 0.102458 | 0.1944027 |
| 17 | 11239 | miRPlus_11239 | Down | 0.2604737 | 0.2095638 |
| 18 | 29190 | hsa-miR-708 | Down | 0.102718 | 0.2338919 |
| 19 | 17354 | hsa-miR-637 | Down | 0.1775424 | 0.250326 |
| 20 | 29529 | hsa-miR-369-3p | Down | 0.1627449 | 0.2519951 |
| 21 | 17652 | hsa-miR-558 | Down | 0.1143609 | 0.2561607 |
| 22 | 27740 | hsa-miR-574-5p | Down | 0.3318467 | 0.2623592 |
| 23 | 10975 | hsa-miR-182 | Down | 0.1616124 | 0.2683256 |
| 24 | 42964 | hsa-miR-7-2* | Down | 0.218878 | 0.2711424 |
| 25 | 5730 | hsa-miR-208a | Down | 0.4340363 | 0.2869522 |
| 26 | 21702 | hsa-miR-219-1-3p | Down | 0.4383205 | 0.2930961 |
| 27 | 42538 | hsa-miR-196a* | Down | 0.159775 | 0.3178579 |
| 28 | 11182 | hsa-miR-98 | Down | 0.4680584 | 0.3198737 |
| 29 | 10952 | hsa-miR-146a | Down | 0.1932844 | 0.3496838 |
| 30 | 10306 | hsa-miR-146b-5p | Down | 0.4813064 | 0.3602452 |
| 31 | 30493 | hsa-miR-548a-5p | Down | 0.4584639 | 0.372827 |
| 32 | 27533 | hsa-miR-320 | Down | 0.1774799 | 0.3807706 |
| 33 | 42994 | hsa-miR-620 | Down | 0.4768922 | 0.3922145 |
| 34 | 42702 | hsa-miR-30c-1* | Down | 0.4289306 | 0.3923156 |
| 35 | 42950 | hsa-miR-24-2* | Down | 0.3219839 | 0.4124028 |
| 36 | 17953 | hsa-miR-183* | Down | 0.2296283 | 0.4166552 |
| 37 | 42856 | miRPlus_42856 | Down | 0.3246721 | 0.4258698 |
| 38 | 10925 | hsa-miR-10b | Down | 0.2899513 | 0.4262885 |
| 39 | 42589 | hsa-miR-509-3-5p | Down | 0.2529337 | 0.4488925 |
| 40 | 11052 | hsa-miR-31 | Down | 0.2421472 | 0.4496652 |
| 41 | 29736 | hsa-miR-656 | Down | 0.310446 | 0.4511076 |
| 42 | 29852 | hsa-miR-9* | Down | 0.4842041 | 0.4760078 |
| 43 | 11023 | hsa-miR-222 | Down | 0.4257719 | 0.4762564 |
| 44 | 42581 | hsa-miR-513a-5p | Down | 0.173074 | 0.4847486 |
| 45 | 42827 | hsa-miR-652 | Down | 0.2664617 | 0.4955641 |
