# Supplementary material for: MiR-218 Inhibits Invasion and Metastasis of Gastric Cancer by Targeting the Robo1 Receptor
Source: PLoS Genet. 2010 Mar 12;6(3):e1000879. doi: 10.1371/journal.pgen.1000879 (PMC2837402; doi:10.1371/journal.pgen.1000879)
Supplement: Table S2 — Clinicopathologic features in 40 tumor samples. (0.25 MB DOC) [file pgen.1000879.s007.doc]

**Table S2.** Clinicopathologic features in 40 tumor samples.

| No | Age (years) | Gender | Tumor size | Depth of tumor invasion | Degree of differentiation | Lymph node status | Distant metastasis | TNM | Stage | Survival (months) | Death^A^ |
| --- | --- | --- | --- | --- | --- | --- | --- | --- | --- | --- | --- |
| T1 | 45 | Male | 2*3*3 cm | T3 | poorly differentiated | N_0_ | M_0_ | T3N0M0 | Ⅱ | 29 | 1 |
| T2 | 26 | Female | 6*6*7 cm | T3 | poorly differentiated | N_1_ | M_0_ | T3N1M0 | Ⅲ | 19 | 1 |
| T3 | 61 | Female | 4*5*5 cm | T3 | poorly differentiated | N_1_ | M_0_ | T3N1M0 | Ⅲ | 26 | 1 |
| T4 | 61 | Female | 2*3*2 cm | T2 | poorly differentiated | N_1_ | M_0_ | T2N1M0 | Ⅱ | 38 | 1 |
| T5 | 52 | Male | 8*7*6 cm | T2 | moderately differentiated | N_2_ | M_0_ | T2N2M0 | Ⅲ | 36 | 0 |
| T6 | 59 | Male | 3*3*3 cm | T2 | moderately differentiated | N_2_ | M_0_ | T2N2M0 | Ⅲ | 27 | 1 |
| T7 | 60 | Male | 4*3*3 cm | T2 | well-differentiated | N_0_ | M_0_ | T2N0M0 | Ⅰ | 61 | 0 |
| T8 | 54 | Male | 5*4*4 cm | T2 | moderately differentiated | N_0_ | M_0_ | T2N0M0 | Ⅰ | 65 | 1 |
| T9 | 28 | Female | 3*3*4 cm | T2 | moderately differentiated | N_0_ | M_0_ | T1N0M0 | Ⅰ | 71 | 0 |
| T10 | 77 | Male | 6*5*6 cm | T2 | poorly differentiated | N_0_ | M_0_ | T2N0M0 | Ⅰ | 61 | 1 |
| T11 | 74 | Male | 5*5*4 cm | T2 | well-differentiated | N_1_ | M_0_ | T2N1M0 | Ⅱ | 36 | 0 |
| T12 | 60 | Male | 8*8*8 cm | T3 | well- and moderately differentiated | N_2_ | M_0_ | T3N2M0 | Ⅲ | 23 | 1 |
| T13 | 70 | Male | 5*5*4 cm | T3 | moderately differentiated | N_1_ | M_0_ | T3N1M0 | Ⅲ | 26 | 1 |
| T14 | 60 | Male | 2*3*3 cm | T2 | poorly differentiated | N_1_ | M_0_ | T2N1M0 | Ⅱ | 43 | 1 |
| T15 | 31 | Male | 4*3*3 cm | T2 | poorly differentiated | N_1_ | M_0_ | T2N1M0 | Ⅱ | 39 | 0 |
| T16 | 59 | Male | 5*4*3 cm | T3 | poorly differentiated | N_1_ | M_0_ | T3N1M0 | Ⅲ | 28 | 1 |
| T17 | 51 | Male | 3*5*4 cm | T2 | poorly differentiated | N_0_ | M_0_ | T2N0M0 | Ⅰ | 60 | 0 |
| T18 | 42 | Male | 8*8*8 cm | T3 | poorly differentiated | N_2_ | M_0_ | T3N2M0 | Ⅲ | 21 | 1 |
| T19 | 44 | Male | 2*2*2 cm | T2 | well-differentiated | N_0_ | M_0_ | T2N0M0 | Ⅰ | 62 | 0 |
| T20 | 52 | Female | 4*5*5 cm | T3 | poorly differentiated | N_2_ | M_0_ | T3N2M0 | Ⅲ | 27 | 1 |
| T21 | 57 | Male | 4*3*3 cm | T2 | moderately differentiated | N_0_ | M_0_ | T2N0M0 | Ⅰ | 61 | 0 |
| T22 | 42 | Female | 4*4*4 cm | T3 | moderately differentiated | N_2_ | M_0_ | T3N2M0 | Ⅲ | 29 | 1 |
| T23 | 58 | Male | 6*8*8 cm | T3 | moderately differentiated | N_2_ | M_1_ | T3N2M1 | Ⅳ | 16 | 1 |
| T24 | 48 | Female | 6*6*5 cm | T4 | poorly differentiated | N_2_ | M_1_ | T4N2M1 | Ⅳ | 13 | 1 |
| T25 | 32 | Male | 3*5*5 cm | T3 | poorly differentiated | N_1_ | M_0_ | T3N1M0 | Ⅲ | 22 | 1 |
| T26 | 72 | Male | 3*3*4 cm | T3 | moderately differentiated | N_1_ | M_0_ | T3N1M0 | Ⅲ | 15 | 1 |
| T27 | 58 | Male | 6*6*5 cm | T3 | moderately differentiated | N_2_ | M_0_ | T3N2M0 | Ⅲ | 38 | 0 |
| T28 | 60 | Male | 8*8*6 cm | T3 | poorly differentiated | N_2_ | M_0_ | T3N2M0 | Ⅲ | 19 | 1 |
| T29 | 60 | Male | 8*7*6 cm | T2 | moderately differentiated | N_2_ | M_0_ | T2N2M0 | Ⅲ | 24 | 1 |
| T30 | 39 | Male | 2*3*3 cm | T3 | poorly differentiated | N_2_ | M_0_ | T3N2M0 | Ⅲ | 37 | 1 |
| T31 | 64 | Male | 4*3*3 cm | T2 | poorly differentiated | N_1_ | M_0_ | T2N1M0 | Ⅱ | 52 | 1 |
| T32 | 48 | Female | 6*6*5 cm | T4 | poorly differentiated | N_2_ | M_1_ | T4N2M1 | Ⅳ | 11 | 1 |
| T33 | 58 | Female | 4*5*3 cm | T1 | poorly differentiated | N_2_ | M_0_ | T1N2M0 | Ⅱ | 32 | 1 |
| T34 | 44 | Female | 5*5*5 cm | T2 | poorly differentiated | N_0_ | M_0_ | T2N0M0 | Ⅰ | 60 | 0 |
| T35 | 69 | Male | 1.5*1.5*2 cm | T2 | moderately differentiated | N_0_ | M_0_ | T1N0M0 | Ⅰ | 41 | 1 |
| T36 | 43 | Male | 8*7*6 cm | T2 | poorly differentiated | N_2_ | M_0_ | T2N2M0 | Ⅲ | 28 | 1 |
| T37 | 65 | Male | 6*5*6 cm | T2 | poorly differentiated | N_2_ | M_0_ | T2N2M0 | Ⅲ | 34 | 1 |
| T38 | 56 | Male | 6*5*5 cm | T2 | moderately differentiated | N_0_ | M_0_ | T2N0M0 | Ⅰ | 63 | 1 |
| T39 | 61 | Male | 6*6*5 cm | T3 | moderately differentiated | N_2_ | M_0_ | T3N2M0 | Ⅲ | 25 | 1 |
| T40 | 65 | Male | 4*6*6 cm | T2 | poorly differentiated | N_1_ | M_0_ | T2N1M0 | Ⅱ | 40 | 0 |
| A: The value is "1" when the subject died at the specified time or "0" when the subject's data was no longer available after that time (the patient was lost to follow-up). | | | | | | | | | | | |
