# Supplementary material for: MiR-218 Inhibits Invasion and Metastasis of Gastric Cancer by Targeting the Robo1 Receptor
Source: PLoS Genet. 2010 Mar 12;6(3):e1000879. doi: 10.1371/journal.pgen.1000879 (PMC2837402; doi:10.1371/journal.pgen.1000879)
Supplement: Table S3 — Primer sequences used in the study. (0.04 MB DOC) [file pgen.1000879.s008.doc]

**Table S3. Primer sequences used in the study.**

| Accession No. | Symbol | Sequence | Product |
| --- | --- | --- | --- |
| NR_003286.1 | 18S rRNA | 5’- GGGCATTCGTATTGCGCCGCTAGAG -3’ | 100 bp |
|  |  | 5’- CGTTCTTGATTAATGAAAACATTCT -3’ |  |
| NM_002941.2 | Robo1 for | 5′-GCGTGCAGTACTAAGGGAACA-3′ | 63 bp |
|  | Robo1 rev | 5′-GGCTTCTTACATGAACATAATGAA-3′ |  |
| NM_004787.1 | Slit2 for | 5'-CGTTTGGAAAATGTGCAGCATAA-3' | 76 bp |
|  | Slit2 rev | 5'-TTCGATTGCTTCTCAACATCAAAGT-3' |  |
| NM_003062.2 | Slit3 for | 5'-CCGCCTAACTACACAGGTGAGCTAT-3′ | 136 bp |
|  | Slit3 rev | 5'-CGCTGTAGCCAGGGACACACT-3′ |  |
| NM_002941.2 | Robo1 3’-UTR for | 5'-CATCCCTGTCTTAACTG-3′ | 229 bp |
|  | Robo1 3’-UTR rev | 5'-AAACGCTTCTCAACAT-3′ |  |
| MI0000295 | miR-218-precursor-F | 5'-CGGGATCCGACCAGTCGCTGCGGGGCTTTCCTTTG  TGCTTGATCTAACCATGTGGTGGAACGATGGAAA-3′ | 127 bp |
|  | miR-218-precursor-R | 5'-CCCAAGCTTTGCAGGAGAGCACGGTGCTTTCCGCG  GTGCTTGACAGAACCATGTTCCGTTTCCATCGTTC-3′ |  |
| MI0000038 | cel-miR-67- precursor-F | 5'-CGGGATCCGATCAAAGATTCGTCGATCCGCTCATTC  TGCCGGTTGTTATGCTATTATCAGATTAAGC-3′ | 116 bp |
|  | cel-miR-67- precursor-R | 5'- CCCAAGCTTAAGTTTTAAAATCGATCTACTCTT  TCTAGGAGGTTGTGATGCTTAATCTGAT-3′ |  |
