# Supplementary material for: MiR-218 Inhibits Invasion and Metastasis of Gastric Cancer by Targeting the Robo1 Receptor
Source: PLoS Genet. 2010 Mar 12;6(3):e1000879. doi: 10.1371/journal.pgen.1000879 (PMC2837402; doi:10.1371/journal.pgen.1000879)
Supplement: Text S1 — Supplementary methods. (0.05 MB DOC) [file pgen.1000879.s009.doc]

**Supplementary Methods**

[**MTT Assay**](http://www.protocol-online.org/cgi-bin/prot/jump.cgi?ID=3254)

Cells were grown in RPMI-1640 medium containing 10% fetal serum. For cell growth measurements, 1×10^3^ cells were seeded in flat-bottom 96-well plates and incubated at 37°C in 5% CO_2_ for 24 h. The MTT working solution was added to the medium, and the cells were incubated for 4 h. The medium was removed, and 150 μl DMSO was added to dissolve the formazan crystals that had formed. Cell viability was measured by absorbance at 490 nm using a microplate reader (Model 680 Microplate Reader, Bio-Rad). The proliferation assay was repeated at least three times.

**Tumorigenicity in nude mice**

Cells were washed and resuspended in PBS. Five-week-old BALB/C nu/nu nude mice obtained from the Shanghai Laboratory Animal Center of China were injected subcutaneously with 1.5x10^6^ cells and were maintained in a sterile animal facility. These mice were then observed for seven weeks for development of tumors. Experiments were performed with the approval of the Institutional Committee for Animal Research and conformed to national guidelines for the care and use of laboratory animals.

**Immunohistochemical analysis.**

A total of 109 specimens, including 40 gastric cancer specimens, the corresponding non-tumor tissues that were also used in the above miR-218 expression analysis, and 29 metastasis tumor tissues were prepared from the archives at the Xijing Hospital. The tissue sections (4 μm thick) were stained with a rabbit anti-Robo1 polyclonal antibody (2.5 ug/ml, NBP1-02805, Novus Biology, USA) and were counterstained with Meyer’s hematoxylin. Robo1 staining was scored according to Clinical Trial Assay recommendations (0–3+). The data were analyzed by Fisher’s exact test, and *P*<0.05 was considered statistically significant.
